# **The Natural Estrogenic Compound Diarylheptanoid (D3): *In Vitro* Mechanisms of Action and *in Vivo* Uterine Responses via Estrogen Receptor** α

**DOI:** 10.1289/ehp.1206122

**Published:** 2013-01-18

**Authors:** Wipawee Winuthayanon, Pawinee Piyachaturawat, Apichart Suksamrarn, Katherine A. Burns, Yukitomo Arao, Sylvia C. Hewitt, Lars C. Pedersen, Kenneth S. Korach

**Affiliations:** 1 Laboratory of Reproductive and Developmental Toxicology, National Institute of Environmental Health Sciences (NIEHS), National Institutes of Health (NIH), Department of Health and Human Services (DHHS), Research Triangle Park, North Carolina, USA; 2 Department of Physiology, Faculty of Science, Mahidol University, Bangkok, Thailand; 3 Department of Chemistry, Faculty of Science, Ramkhamhaeng University, Bangkok, Thailand; 4 Laboratory of Structural Biology, NIEHS, NIH, DHHS, Research Triangle Park, North Carolina, USA

**Keywords:** diarylheptanoid, ER-dependent, nuclear translocation, phytoestrogen, uterus

## Abstract

Background: Diarylheptanoid (D3) isolated from the medicinal plant, *Curcuma comosa*, has estrogenic activity.

Objective: We aimed to elucidate the mechanism(s) of D3 action and compare it with that of 17β-estradiol (E_2_) using both *in vitro* and *in vivo* uterine models.

Methods: We used human uterine (Ishikawa) cells to determine the estrogenic action of D3 on the activation and nuclear translocation of estrogen receptor α (ERα). In addition, we further characterized the uterine response to D3 treatment *in vivo*.

Results: D3 activated an estrogen responsive element (ERE) luciferase reporter through ERα, and molecular modeling suggested that D3 could be accommodated in the ERα binding pocket. Using modified ERα to assay ligand-dependent nuclear translocation, we observed D3-dependent ERα interaction and translocation. In mouse uteri, early- and late-phase estrogen-regulated gene responses were increased in D3-treated ovariectomized wild-type animals, in a manner similar to that of E_2_; no response was seen in ERα knockout animals. We observed a divergence in estrogen responses after D3 treatment: D3 induced robust DNA synthesis in uterine epithelial cells, linked to an increase in cell-cycle–related genes; however, no increase in uterine weight was observed 24 hr after treatment. D3 also affected uterine progesterone receptor expression patterns similar to E_2_. When D3 and E_2_ were administered together, we observed no additive or antagonistic effects of D3 on E_2_. Our findings suggest that D3 is a weak estrogenic agonist compound.

Conclusion: D3 is a weakly acting phytoestrogen that mimics the mitogenic responses produced by E_2_ in an ERα-dependent manner, but it is unable to increase uterine weight or enhance or antagonize the effects of estrogen.

Estrogens play important roles in growth, differentiation, and maintenance functions of many target tissues in the female reproductive organs (Couse and Korach 1999). The biological actions of estrogen are mediated primarily through estrogen receptor (ER) α and β (Couse et al. 1997). ERs are members of the nuclear receptor family of proteins containing multiple functional domains: The A/B domain harbors activation function 1 (AF1); the DNA binding domain is located in the C region of the receptors; the hinge region (D domain) contains nuclear localization sequences (NLS) (Mader et al. 1993); and the E/F domains contain the ligand binding region and AF2 function. AF1 and AF2 portions of the protein facilitate transcriptional activity of the ER (Tora et al. 1989). Upon binding ligand, the ER is localized to the nucleus and initiates gene transcription through multiple pathways, including classical estrogen responsive element (ERE)–dependent pathways and nonclassical pathways (Hall et al. 2001).

The uterus is one of the most prominent estrogenic responsive target tissues, predominantly expressing ERα (Couse et al. 1997). Uterine response to estrogen is rapid and eventually leads to a dramatic increase in cell proliferation (Martin et al. 1973). However, the uterotrophic responses to estrogen vary with time after hormone exposure. An early response of water imbibition in uteri is mediated through ERα; ERα knockout (αERKO) mice show no water imbibition and no increase in uterine weight after 17β-estradiol (E_2_) treatment (Korach 1994). The genomic responses of the uterus to E_2_ have been observed 0.5–96 hr after treatment (Hewitt et al. 2003; Naciff et al. 2007). Some exogenous estrogens (bisphenol A and genistein), as well as one of the endogenous estrogens (estriol), are considered weak estrogens in the uterus. Weak estrogenic compounds are less potent than E_2_; they exhibit early uterine responses but are less effective in their abilities to cause robust subsequent uterine responses such as cellular hypertrophy and hyperplasia (Hewitt and Korach 2011). Stronger estrogens, including E_2_, initiate both early and late effects (Anderson et al. 1975). Transcripts that increase 1–2 hr after acute dosing of estrogenic compounds are components of the E_2_ responsive “early gene cluster,” which includes *Fos* and *Inhbb* (inhibin beta-B) (Hewitt et al. 2003). The late responses include increased and sustained RNA and protein synthesis, which lead to uterine cellular hypertrophy, DNA synthesis, and hyperplasia (Hewitt et al. 2003), as well as an alteration of progesterone receptor (PR) expression patterns (Mote et al. 2006). A second response phase is characterized by a wave of mitosis and DNA synthesis, which occurs 16–24 hr after E_2_ treatment and is correlated with the late-phase cell cycle regulators, including *Aurkb* (aurora kinase B) and *Ccnb2* (cyclin B2) (Hewitt et al. 2003; Hewitt and Korach 2011). The early and late events reflect the uterotrophic action of estrogens on uterine tissues and have been widely used to evaluate and compare potency and estrogenic or antagonistic activity of xenoestrogenic compounds.

Diarylheptanoids are phytoestrogens isolated from *Curcuma comosa,* a plant in the Zingiberaceae family. *C. comosa* has been marketed as a plant-derived dietary supplement product traditionally used in indigenous medicine as an alternative remedy for hormone replacement therapy in menopausal women (Piyachaturawat et al. 1995). Other diarylheptanoids are found in *Curcuma* and other plants in the ginger family (Keserü and Nógrádi 1995). D3 (Figure 1A), one of the most abundant purified diarylheptanoids from *C. comosa* rhizome extract (Suksamrarn et al. 2008), exerts the most potent estrogenic activity when administered for 2 or 3 consecutive days in a rodent uterine bioassay (Winuthayanon et al. 2009a, 2009b). D3 has also been reported to have a vascular relaxative effect in the endothelial cells of rat aortic rings, similar to the effect of estrogen (Intapad et al. 2012). These biological actions of D3 may potentially benefit women without causing adverse side effects such as those caused by current or traditional estrogen replacement therapy (Shifren and Schiff 2010). Because of the high availability of D3 (Suksamrarn et al. 2008), the estrogenic-like bioactivities of D3, and the long-term favorable use of these plant products by daily consumption (in the form of dried fine rhizome power in capsules or as decoctions twice a day), we aimed to characterize the *in vitro* and *in vivo* mechanism(s) of action of D3, focusing on its effect in uterine cells. We evaluated the estrogenic activities of D3 on wild-type (WT) and ERα-mutant receptor in a human uterine (Ishikawa) cell line as well as evidence of D3 binding to the ERα using a new cellular assay for detecting direct interaction of D3 to the ERα. In addition, we evaluated both early and late biological responses in the mouse uterus, including any potential effect on modulating the action of E_2_. This work indicates that—in both a human uterine cell model and in the rodent uterus—D3 has weak estrogenic activity that is mediated through ERα, and that D3 does not synergize or antagonize the effects of E_2_.

**Figure 1:**
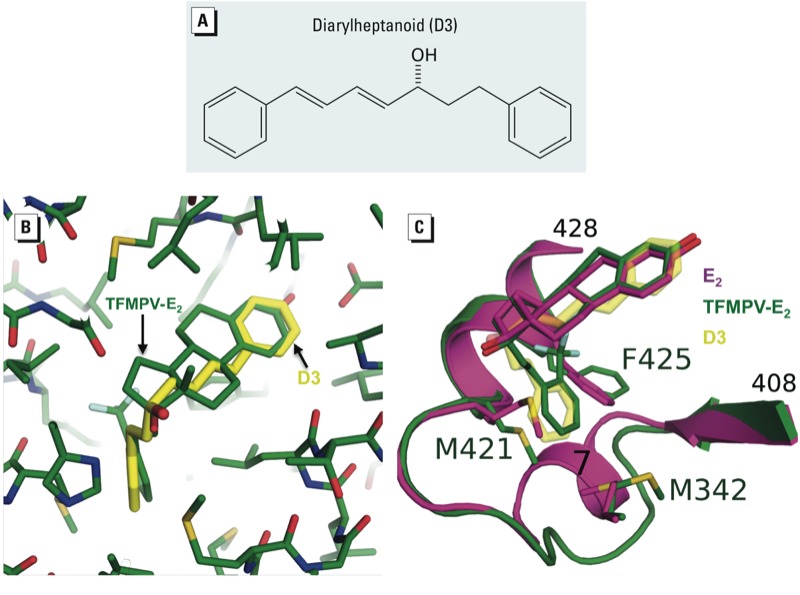
Potential binding mode of D3 to ERαLBD. (*A*) Chemical structure of D3. (*B*) Superimposition of the D3 model (yellow) onto the TFMPV-E_2_ agonist (green) bound to the ERαLBD. (*C*) Superimposition based on the crystal structures of E_2_ bound to ERαLBD (magenta) and TFMPV-E_2_ bound to the ERαLBD (green) reveals the conformational changes between the binding modes of the two agonists. Helix 7 unwinds in the TFMPV-E_2_ binding mode, and side chains M342, M421, and F425 all have altered conformations to accommodate the E_2_ ligand substitution. D3 is superimposed (yellow). (*B*) and (*C*) were created using PyMol, version 1.4 (Schrodinger, LLC; http://www.pymol.org).

## Methods

*Chemicals*. We purchased E_2_ from Sigma (St. Louis, MO, USA) and ICI 182,780 (ICI) from Tocris Bioscience (Ellisville, MS, USA). All chemicals were dissolved in ethanol unless otherwise indicated. D3 [(3*R*)-1,7-diphenyl-(4*E*,6*E*)-4,6-heptadien-3-ol; Figure 1A] was isolated from *C. comosa* as described previously (Suksamrarn et al. 2008).

*Three-dimensional modeling of D3*. The model for D3 was created using Insight II, version 2005 (Accelrys Inc., San Diego, CA, USA) and minimized using the Discover_3 force field. The model was manually superimposed onto the structure of trifluoromethylphenylvinyl estradiol (TFMPV-E_2_) in the crystal structures of TFMPV-E_2_ bound to the ligand-binding domain (LBD) of ERα [ERαLBD; Protein Data Bank (PDB) 2P15 (Nettles et al. 2007)] and E_2_ bound to ERαLBD [PDB 1GWR (Wärnmark et al. 2002)]; this was followed by additional minimization of the ligand docked to the crystal structure of P215 to relieve any significant strain that may have been created from the manual modeling.

*Plasmids*. We used the expression plasmids for mouse pcDNA3-WT-ERα (WT ERα; Winuthayanon et al. 2009a) and pcDNA3-H2-ERα [D-domain ERα mutant; Hinge 2 (H2) ERα], the disrupted NLS mutant of ERα, and pcDNA3-H2-ERα-EGFP [D-domain ERα mutant with green fluorescent protein (GFP) fused; H2 ERα-GFP] (Burns et al. 2011). The H2 ERα has a modified nuclear localization sequence, so the H2 ERα remains predominantly localized in the nonnuclear compartment in the absence of ligand, and translocates to the nucleus when bound and interacting with the ligand (Burns et al. 2011). The 3× ERE-TATA-Luc (luciferase)–expressing plasmid was a gift from D. McDonnell (Duke University Medical Center, Durham, NC, USA). pRL-tk (constitutively expressed renilla) was purchased from Promega (Madison, WI, USA).

*Cell culture and transfection conditions*. Human endometrial adenocarcinoma (Ishikawa) cells that do not express endogenous ER (ER-negative) were a gift from R. DiAugustine [National Institute of Environmental Health Sciences (NIEHS)] (Ignar-Trowbridge 1993). HeLa human cervical epithelial cells were purchased from ATCC (Manassas, VA, USA). Cell culture reagents were purchased from Invitrogen Life Technologies (Invitrogen, Carlsbad, CA, USA) unless otherwise indicated. Cell culture conditions were described previously [Winuthayanon et al. 2009a; for additional information, see Supplemental Material, p. 3 (http://dx.doi.org/10.1289/ehp.1206122)].

*Confocal microscopy*. HeLa cells were used for the GFP-tagged H2 ERα translocation experiment because of their high transfection efficiency. HeLa cell culture and treatment conditions were previously described by Burns et al. (2011). Briefly, HeLa cells were plated on Lab-Tek 2-well chamber slides (NUNC, Rochester, NY, USA) overnight. Cells then were transfected with 0.4 μg of H2 ERα-GFP in Dulbecco’s modified Eagle medium supplemented with 10% dextran-coated charcoal–stripped fetal bovine serum for 8 hr. At 27 hr after the transfection, cells were treated for 3 hr with ethanol (vehicle), E_2_ (10 nM), or D3 (50 μM). Cells were then fixed and visualized on a Zeiss 510-UV meta confocal microscope (Carl Zeiss, Inc., Thornwood, NY, USA) to determine cellular localization of H2 ERα-GFP, as previously described (Burns et al. 2011). The cellular colocalization of H2 ERα-GFP and DAPI (for nucleus) was quantified with the Multi Wavelength Cell Scoring application from MetaMorph Microscopy Automation and Image Analysis Software (version 7.7.0.0; Molecular Devices, Downington, PA, USA).

*Uterine bioassay in adult WT ovariectomized mice*. Animals were handled according to NIEHS Animal Care and Use Committee guidelines and in compliance with an NIEHS-approved animal protocol. The animals were treated humanely and with regard for alleviation of suffering. Adult female C57BL/6J mice (8 weeks of age) were purchased from Charles River Laboratories (Raleigh, NC, USA). C57BL/6J αERKO mice (Lubahn et al. 1993) were generated at Taconic Farms (Germantown, NY, USA). All mice were ovariectomized (OVX) and held for 2 weeks to recover and eliminate endogenous ovarian steroids before the study. Mice were randomly grouped and treated for 2 or 24 hr with sesame oil [vehicle; subcutaneous (sc) administration], D3 (100 mg/kg) dissolved in 100 µL sesame oil (sc), or E_2_ (10 µg/kg) dissolved in 100 µL saline [intraperitoneal (ip) administration). In some experiments, WT OVX animals were treated with both D3 (100 mg/kg) and E_2_ (10 µg/kg). To measure DNA synthesis for the 24-hr time point, EdU (5-ethynyl-2´-deoxyuridine; 2 mg/mL in 100 µL phosphate-buffered saline) was delivered as a second injection (ip) 2 hr prior to tissue collection (22 hr after vehicle, E_2_, or D3 injection). Animals were euthanized by CO_2_ asphyxiation. Tissue collection and real-time polymerase chain reaction (PCR) were performed as described previously [Winuthayanon et al. 2010; for additional information, see Supplemental Material, pp. 3–4 (http://dx.doi.org/10.1289/ehp.1206122)].

*Statistical analysis*. The results are expressed as mean ± SE. The statistical difference among groups was compared using one-way analysis of variance (ANOVA) followed by Tukey’s post test, or by two-way ANOVA followed by Bonferroni’s posttest. Statistical significance was considered at *p* < 0.05.

## Results

*Modeling of D3 to ER*α *supports agonist binding*. To model potential D3 binding to ERα, we used three-dimensional molecular docking. The structure of E_2_ bound to the ERα LBD does not indicate how D3 could behave as an agonist. Because D3 is a larger molecule than E_2_, there does not appear to be enough space in the ERα binding pocket to accommodate D3 binding. However, the crystal structure of the potent ERα agonist TFMPV-E_2_ bound to ERα suggests flexibility and conformational changes in the binding pocket, allowing accommodation of the bulkier TFMPV-E_2_ ligand (Nettles et al. 2007), specifically the unwinding of helix 7 and the alteration of the side chains M342, M421, and F425 (Figure 1B–C). Superimposing D3 onto TFMPV-E_2_ and then minimizing the ERα binding pocket suggests that ERα could potentially accommodate D3 in an agonist-type binding mode similar to that when TFMPV-E_2_ is bound (Figure 1C).

**Figure 2:**
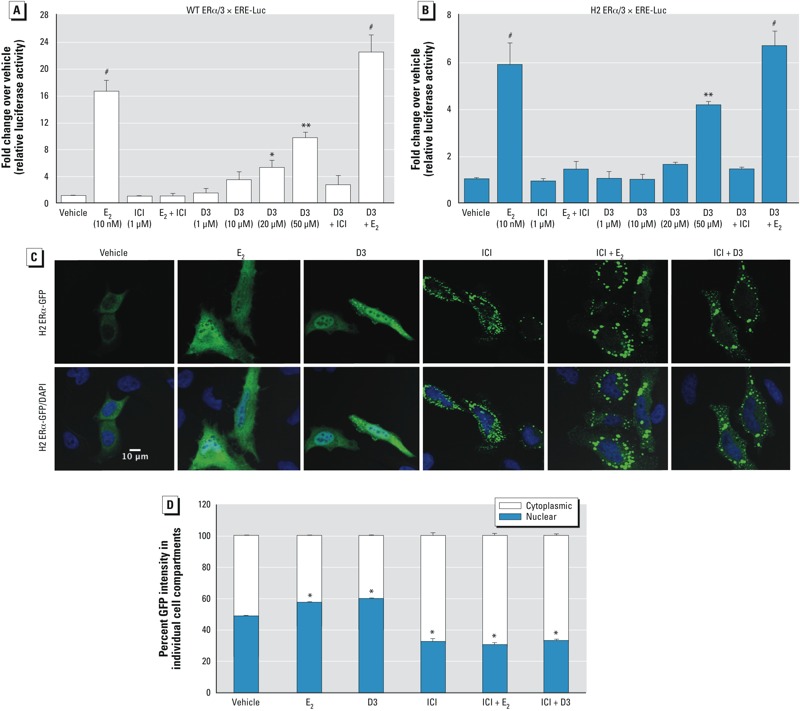
D3 transactivated and induced the shuttling of ERα from the cytoplasm into the nucleus. (*A, B*) D3 dose–response curve of luciferase activity in Ishikawa cells in the presence of mouse WT ERα (*A*) and H2 ERα mutant (*B*) after 24‑hr treatment with ethanol vehicle, E_2_, ICI, ICI + E_2_, D3, ICI + D3 (50 µM), or D3 (50 µM) + E_2_. Values represent the mean ± SE of triplicates from three experiments. (*C*) H2 ERα-GFP expression in each cell compartment after treatment with vehicle, E_2_ (10 nM), D3 (50 µM), ICI (1 µM), ICI (1 µM) + E_2_ (10 nM), or ICI (1 µM) + D3 (50 µM) for 3 hr. The translocation of H2 ERα-GFP (green) from the cytoplasmic to nuclear compartment was visualized by confocal microscopy. DAPI (blue) was used to visualize the cell nucleus. All images represent the same magnification; bar = 10 µm. (*D*) Quantification of H2 ERα-GFP colocalization. The signal from H2 ERα-GFP and DAPI were analyzed as a percentage within each cell compartment compared with whole-cell intensity (100–180 cells/group); values are mean ± SE. *p < 0.05, **p < 0.01, and ^#^*p* < 0.001 compared with vehicle treatment.

*D3 activates ER*α*-dependent transcription*. Our previous study in liver cancer (HepG2) cell lines and molecular modeling suggested that D3 could act as an agonist with ERα (Winuthayanon et al. 2009a). We further evaluated the mechanism of D3 on ERα-mediated transcriptional activity *in vitro* in the uterine cell model. Plasmids containing WT ERα and 3 × ERE-Luc were transiently transfected into Ishikawa cells. In the presence of WT ERα, 10 nM E_2_ significantly (*p* < 0.001) increased luciferase activity compared with the vehicle control, and E_2_-induced transcription was fully inhibited by ICI, an ER antagonist (Figure 2A). Compared with vehicle, D3 significantly stimulated ERE-dependent luciferase activity in a dose-dependent manner, with the maximum luciferase activity at doses of 20 and 50 µM D3 (*p* < 0.05 and *p* < 0.01, respectively). The co-treatment of D3 with ICI inhibited ERE-dependent luciferase activity. No statistical differences were observed between E_2_-treated and D3+E_2_-treated groups, suggesting that D3 did not exhibit antagonism or alter E_2_-induced transcription.

*D3 interacts with and translocates ER*α *to the nucleus*. In the transfection studies, WT ERα is primarily located in the nucleus, even in the absence of estrogen ligand (Burns et al. 2011). We used H2 ERα as a tool to assess the ability of D3 to initiate direct ERα interaction, transactivation, and translocation as a measure of D3–ERα interaction. Both E_2_ (10 nM) and D3 (50 µM) significantly induced 3 × ERE-Luc in the presence of H2 ERα (Figure 2B; *p* < 0.001 and 0.01, respectively). The transactivation activity induced by either E_2_ or D3 is fully inhibited by ICI; the co-treatment of D3 with E_2_ did not alter the transactivation induced by E_2_.

Because WT ERα is localized in the nucleus in the absence of ligand, we were unable to illustrate that D3 induced nuclear translocation using WT ERα. Therefore, we used H2 ERα-GFP transfected into HeLa cells to test D3 binding by visualizing that D3 increases the translocation of ERα to the nucleus. The D3 treatment caused increased H2 ERα-GFP signal in the nuclei, similar to that of E_2_ treatment (Figure 2C). To illustrate that the nuclear translocation induced by D3 is ERα-dependent, we co-treated D3 with ICI. ICI treatment alone induced a punctate pattern in the cytoplasm reminiscent of protein degradation, which is known to occur for ER with ICI treatment (Dauvois et al. 1993). Nuclear translocation of H2 ERα-GFP by treatment with D3 or E_2_ was disrupted by ICI co-treatment. Results indicate that D3 action was mediated through ERα interaction. Quantitated nuclear and cytoplasmic H2 ERα-GFP intensities demonstrated that E_2_ and D3 treatment resulted in a significant increase in the percentage of H2 ERα-GFP intensity in the nucleus compared with vehicle treatment (*p* < 0.05; Figure 2D). ICI treatment—either alone or with ligands—resulted in a higher percentage of cytoplasmic H2 ER-GFP intensity. Collectively, D3 induced ERα-interaction, translocation, and nuclear occupancy; thus D3 is able to mediate ERα activity.

**Figure 3:**
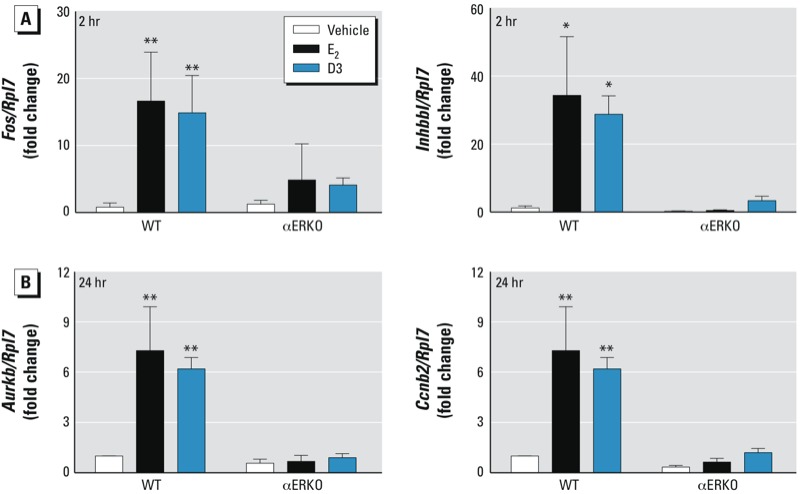
D3 stimulates ERα-dependent early and late gene responses in the mouse uterus. Early (2 hr; *Fos* and *Inhbb*; *A*) and late (24 hr; *Aurkb* and *Ccnb2*; *B*) transcripts in the uterus after the treatment with vehicle (sesame oil), E_2_ (10 µg/kg), or D3 (100 mg/kg) in adult OVX WT and αERKO mice. Values represent mean ± SE (*n* = 4). *p < 0.05, and **p < 0.01 compared with vehicle treatment within the genotype.

*D3 stimulates an ER*α*-dependent response in the uterus*. Because our results suggest that D3 utilizes ERα and induces ERE-dependent transcription in a manner similar to that of E_2_ *in vitro*, we evaluated the transcriptional profile of D3 compared with E_2_ in an *in vivo* uterine model using WT and αERKO animals. The physiological responses of the mouse uterus to E_2_ consist of both early- and late-phase events (Hewitt et al. 2003). Thus, we examined the effects of D3 on the early (2 hr) and late (24 hr) events in OVX mice. We used a D3 dose of 100 mg/kg, a dose previously shown to exert maximal uterine responses (Winuthayanon et al. 2009a). At 2 hr, the E_2_-regulated genes *Fos* and *Inhbb* were significantly up-regulated in WT mice treated with either E_2_ or D3 (*p* < 0.01 for *Fos*, and *p* < 0.05 for *Inhbb*) compared with vehicle (Figure 3A). *Aurkb* and *Ccnb2* were also significantly up-regulated in WT uteri after E_2_ or D3 treatment at 24 hr (*p* < 0.01) (Figure 3B). We observed no gene activation at 2 or 24 hr in uteri from either E_2_- or D3-treated αERKO mice, indicating the requirement of ERα for early and late response activation by E_2_ and D3.

**Figure 4:**
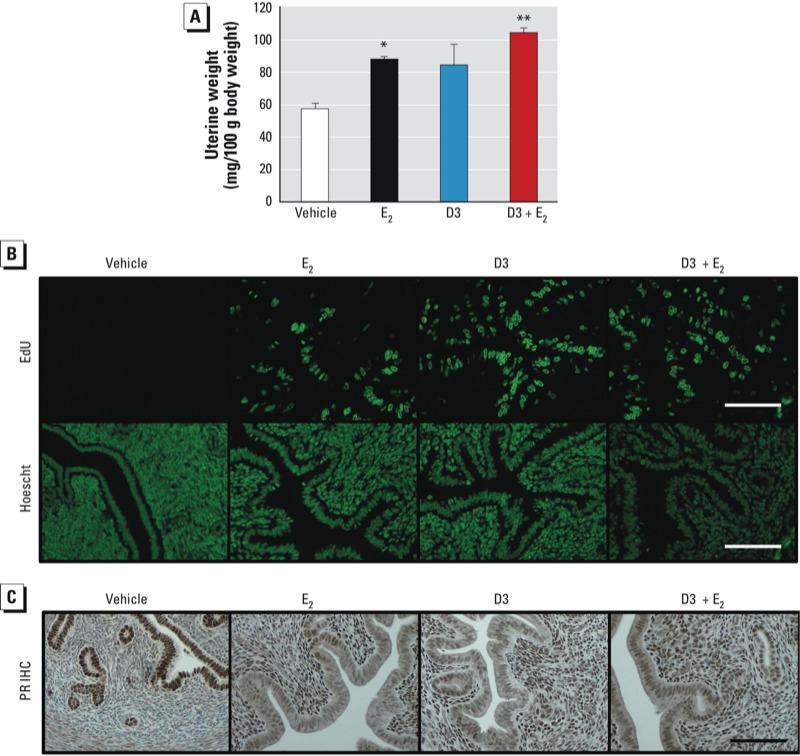
D3 induced uterine DNA synthesis, but not uterine weight increase, in adult mice, with no additive or antagonistic effects on E_2_ treatment. Adult OVX females were treated for 24 hr with vehicle (sesame oil), E_2_ (10 µg/kg), D3 (100 mg/kg), or D3 (100 mg/kg) plus E_2_ (10 µg/kg). (*A*) Normalized uterine weight; values are mean ± SE (*n* = 3–4). (*B*) DNA synthesis in uterine luminal and glandular epithelium shown by EdU incorporation (S-phase of DNA synthesis) and Hoescht 33342 (DNA staining). (*C*) Immunohistochemistry showing PR protein expression in uterine sections. Representative images are shown; bars = 100 µm. *p < 0.05, and **p < 0.01 compared with vehicle treatment.

*D3 does not alter estrogen action in the uterus*. Estrogenic action of D3 in the uterus was ERα-dependent; therefore, focusing on the responses in WT animals, we evaluated uterine wet weight increase, epithelial cell proliferation, and PR expression patterns as parameters of biological responses at 24 hr. Biological responses were also assessed in the presence or absence of E_2_ (10 µg/kg) to determine whether D3 would exhibit antiestrogenic activity in the uterus. As we expected, E_2_ treatment significantly increased uterine wet weight (*p* < 0.05); however, D3 (100 mg/kg) did not (Figure 4A). Co-treatment of D3 with E_2_ neither augmented nor diminished the E_2_-induced increase in uterine wet weight. Although uterine weight was not significantly induced by D3 treatment, D3 did induce uterine DNA synthesis as shown by the positive signal of EdU incorporation in uterine epithelial cells, similar to that of E_2_ (Figure 4B). D3 plus E_2_ did not alter the level of DNA synthesis in uterine epithelium above the level induced by E_2_ alone. To evaluate estrogen responsiveness, we evaluated PR protein expression patterns by immunohistochemical analysis. PR was expressed in the uterine luminal and glandular epithelium in the absence of ovarian hormones (after OVX), as observed in vehicle-treated animals (Figure 4C). In the presence of E_2_, PR expression decreased in the uterine epithelium but increased in the uterine stroma (Figure 4C; see also Tibbetts et al. 1998). In a manner similar to E_2_, D3 decreased PR expression in the uterine epithelium and increased PR in the stroma. The PR expression pattern for D3 plus E_2_ was similar to that of E_2_ alone, indicating that D3 has weak estrogenic agonist activity and does not exert antiestrogenic effects on PR expression in the uterus.

## Discussion

We previously showed that D3, a naturally occurring phytoestrogenic compound from *C. comosa*, exhibited estrogen-like activity *in vitro* and *in vivo* (Suksamrarn et al. 2008; Winuthayanon et al. 2009a, 2009b); however, the underlying mechanism(s) of uterine action of D3 had not been investigated. In the present study, we further characterized the mechanisms of the uterotrophic responses of D3 in human uterine cells, as well as in an animal model, for comparison with an endogenous hormone, E_2_. Certain goals of this study were to more clearly understand the mechanism of action of this compound because it shows divergent estrogenic activity, and to clarify the implications of local use of this indigenous plant in women as a health promotional supplement and as an alternative treatment for postmenopausal symptoms. We focused on the transcriptional regulation mediated by ERα in a human uterine cell line and on the profile of different physiological events in uterine responsiveness during proliferation [early (2 hr) and late (24 hr) responses]. We also explored the possible binding mode of D3 to the ERαLBD via molecular modeling.

Historically, compounds with agonist cores and large bulky side chains have behaved as antagonists to the ER by displacing helix 12 from the agonist binding position (Brzozowski et al. 1997). Thus, because of the structural properties of TFMPV-E_2_, it was surprising when Nettles et al. (2007) reported that TFMPV-E_2_ could function as a potent agonist. The crystal structure of TFMPV-E_2_ bound to ERα revealed plasticity in the ERαLBD, whereby the trifluoromethylphenylvinyl side chain could be accommodated by the unwinding and displacement of helix 7 and the rearrangement of a few side chains (Nettles et al. 2007). Binding in this manner increased the volume of the binding pocket by 40% while maintaining helix 12 in a position consistent with agonist binding. Interestingly, the structure of D3 can be reasonably superimposed onto the structure of TFMPV-E_2_ bound to the ERαLBD (Figure 1B). The structure of D3 can be manipulated such that both phenyl groups superimpose with the phenol and phenyl groups of TFMPV-E_2_. Binding in this orientation also positions D3’s hydroxyl oxygen in a similar location to that of the 17β-hydroxyl of E_2_. Although it is unclear whether this is indeed D3’s mode of binding to the ERαLBD, this similiarity does support the possibility that D3 can bind in an orientation consistent with agonist binding and activity. In addition to our modeling, our previous findings using reporter assays in HepG2 cells indicated that the AF2 domain within the ERαLBD is crucial for D3 transcriptional activity, as mutations in the AF2 domain blunted D3 mediated transcriptional responses (Winuthayanon et al. 2009b).

Estrogens exert their regulatory potential on gene expression in target tissues by different mechanisms. Several compounds are able to interact with both ERα and ERβ (Kuiper et al. 1998). The uterus is one of the most estrogen-responsive reproductive tissues that predominantly expresses ERα (Couse et al. 1997; Nilsson et al. 2001). The ligand–ER complex in the nucleus interacts with both ERE or non-ERE (tethered) sequences (Couse and Korach 1999). We previously reported that D3 transactivated genes in an ERα/ERE-dependent manner in human liver cells, with no tethering activity (Winuthayanon et al. 2009a). In the present study we further investigated the mechanisms of action of D3 in uterine cells by introducing WT or H2 ERα in Ishikawa cells. In Ishikawa cells, D3 activated an ERα/ERE-mediated luciferase reporter. However, to obtain a detectable biological response, D3 must be administered at a very high dose compared to E_2_. Traditional ^3^H[E_2_] ligand binding assays using uterine cytosolic preparations were unable to demonstrate D3 binding to ERα [see Supplemental Material, Figure S1 (http://dx.doi.org/10.1289/ehp.1206122)]. This may be due to the very low binding affinity of D3 to ERα, as shown by the high dose required for both reporter gene activity and uterine bioassay, or it may result from use of the crude cytosolic preparation containing binding proteins that may bind nonspecifically to D3, preventing interaction with ERα. Therefore, we used H2 ERα, a mutant that exhibits hormone-dependent translocation from the cytoplasm into the nucleus in the presence of ligand (Burns et al. 2011). In the present study, we observed that D3 treatment induced H2 ERα transactivation in the luciferase reporter assay and in H2 ERα-GFP translocation into the nucleus. Both findings suggest that D3 interacts directly with ERα. We also illustrated that H2 ERα could be a useful and sensitive experimental tool for compounds that exerted weak estrogenic activity and that could not be tested by the conventional ligand binding assay.

Endocrine-disrupting compounds, such as bisphenol A (BPA) and 2,2-bis(*p*-hydroxyphenyl)-1,1,1-trichloroethane (HPTE), are considered “weak estrogens,” exhibiting early-phase estrogenic responses in the uterus, but late responses are diminished after 24 hr (Hewitt and Korach 2011). Similarly, we found that D3 stimulated the expression of early-phase genes (*Fos* and *Inhbb*) in a fashion similar to that of E_2_. In contrast to BPA or HPTE, D3 also sustained its effect on the induction of late-phase genes (*Aurkb* and *Ccnb2*). The transcriptional responses by D3 were mediated through ERα as shown by the lack of gene stimulation in αERKO uteri. In addition to the genomic responses, D3 clearly stimulated DNA synthesis selectively in uterine luminal and glandular epithelium, concomitant with the up-regulation of cell cycle–related genes, such as *Aurkb* and *Ccnb2* (at 24 hr). However, uterine wet weight did not significantly increase with D3 treatment at 24 hr. One explanation for this discrepancy between the tissue response and gene activation is that a repetitive treatment with weak estrogens is required to induce the increase in uterine weight, but changes in gene expression may indicate stimulation of tissues leading to tissue-response end points. In previous studies, uterine weight was significantly increased after treatment with D3 for 2 and 3 consecutive days in immature OVX rats (Winuthayanon et al. 2009b) and adult OVX mice (Winuthayanon et al. 2009a), respectively. However, the significant uterine weight increase induced by D3 was still lower than that induced by E_2_, which is consistent with the property of a weak estrogen. Thus, repeated treatment with D3 is required for the weight-increase response; however, uterine genomic responses of D3 can be observed 2 and 24 hr after a single injection. In addition, the gene expression pattern at 24 hr was sustained by D3 treatment, supporting its potential potency. We also found that, in the presence of the endogenous estrogen E_2_, D3 did not alter the PR expression pattern induced by E_2_. The dose administered *in vivo* in the present study was 100 mg/kg (2.5 mg per mouse). From pharmacokinetic studies in rats, the bioavailability of D3 via oral administration is approximately 24.01% (Su et al. 2012). If mice administered D3 by ip injection bioavailability similar to that in rats, D3 at 100 mg/kg would have a circulating level of D3 of approximately 132.3 µM. This suggests that the dose of D3 used in the *in vivo* experiments would be similar to the dose used *in vitro*. In summary, D3 acts as a weak estrogen through ERα, as shown in both *in vitro* and *in vivo* biological assays.

## Conclusions

We found that the biological actions of D3 were mediated by its transcriptional activity as an agonist for ERα through an ERE-dependent reporter in uterine cells, and that, in mouse uterus, D3 produces uterine responses in both the early and late phases, in a manner similar to that of E_2_, without interfering with the effect of endogenous estrogens. Surprisingly, we also observed that D3 had a unique chemical structure that could be accommodated in the binding pocket of ERα. Our three-dimensional modeling may shed light on how other nonsteroidal endocrine-disrupting compounds exert estrogenic activity through ERα. D3 shows promise as a naturally isolated weak estrogenic compound that might be used as an alternative therapy for symptoms in women that result from estrogen withdrawal. However, either *in vitro* or *in vivo,* D3 must be administered frequently at extremely high doses to produce maximal biological responses that approach—but never equal—E_2_ responses. The identification and characterization of D3’s actions on molecular targets advance our basic knowledge of the phytoestrogen D3’s actions in uterine cells in the presence of the endogenous hormone E_2_. In addition, this study suggests that although D3 acted as a weak agonist, it did not interfere or antagonize the action of E_2_ in the *in vivo* model, which may suggest the use of this plant in ovarian cycling women. Although diarylheptanoids are naturally occurring compounds abundant in spices and vegetables, the possibility of D3’s proliferative DNA synthesis activity and increased risk for cancer should not be overlooked during long-term consumption.

## Supplemental Material

(360 KB) PDF

## References

[r1] Anderson JN, Peck EJ, Clark JH (1975). Estrogen-induced uterine responses and growth: relationship to receptor estrogen binding by uterine nuclei.. Endocrinology.

[r2] Brzozowski AM, Pike AC, Dauter Z, Hubbard RE, Bonn T, Engström O (1997). Molecular basis of agonism and antagonism in the oestrogen receptor.. Nature.

[r3] Burns KA, Li Y, Arao Y, Petrovich RM, Korach KS (2011). Selective mutations in estrogen receptor α D-domain alters nuclear translocation and non-estrogen response element gene regulatory mechanisms.. J Biol Chem.

[r4] Couse JF, Korach KS (1999). Estrogen receptor null mice: what have we learned and where will they lead us?. Endocr Rev.

[r5] Couse JF, Lindzey J, Grandien K, Gustafsson JA, Korach KS (1997). Tissue distribution and quantitative analysis of estrogen receptor-α (ERα) and estrogen receptor-β (ERβ) messenger ribonucleic acid in the wild-type and ERα-knockout mouse.. Endocrinology.

[r6] Dauvois S, White R, Parker MG (1993). The antiestrogen ICI 182780 disrupts estrogen receptor nucleocytoplasmic shuttling.. J Cell Sci.

[r7] Hall JM, Couse JF, Korach KS (2001). The multifaceted mechanisms of estradiol and estrogen receptor signaling.. J Biol Chem.

[r8] Hewitt SC, Deroo BJ, Hansen K, Collins J, Grissom S, Afshari CA (2003). Estrogen receptor-dependent genomic responses in the uterus mirror the biphasic physiological response to estrogen.. Mol Endocrinol.

[r9] Hewitt SC, Korach KS (2011). Estrogenic activity of bisphenol A and 2,2-bis(*p*-hydroxyphenyl)-1,1,1-trichloroethane (HPTE) demonstrated in mouse uterine gene profiles.. Environ Health Perspect.

[r10] Ignar-Trowbridge DM, Teng CT, Ross KA, Parker MG, Korach KS, McLachlan JA (1993). Peptide growth factors elicit estrogen receptor-dependent transcriptional activation of an estrogen-responsive element.. Mol Endocrinol.

[r11] Intapad S, Saengsirisuwan V, Prasannarong M, Chuncharunee A, Suvitayawat W, Chokchaisiri R (2012). Long-term effect of phytoestrogens from *Curcuma comosa* Roxb. on vascular relaxation in ovariectomized rats.. J Agric Food Chem.

[r12] Keserü GM, Nógrádi M (1995). The chemistry of natural diarylheptanoids.

[r13] Korach KS (1994). Insights from the study of animals lacking functional estrogen receptor.. Science.

[r14] Kuiper GGJM, Lemmen JG, Carlsson B, Corton JC, Safe SH, van der Saag PT (1998). Interaction of estrogenic chemicals and phytoestrogens with estrogen receptor β.. Endocrinology.

[r15] Lubahn DB, Moyer JS, Golding TS, Couse JF, Korach KS, Smithies O (1993). Alteration of reproductive function but not prenatal sexual development after insertional disruption of the mouse estrogen receptor gene.. Proc Natl Acad Sci USA.

[r16] Mader S, Chambon P, White JH (1993). Defining a minimal estrogen receptor DNA binding domain.. Nucleic Acids Res.

[r17] Martin L, Finn CA, Trinder G (1973). Hypertrophy and hyperplasia in the mouse uterus after oestrogen treatment: an autoradiographic study.. J Endocrinol.

[r18] Mote PA, Arnett-Mansfield RL, Gava N, deFazio A, Mulac-Jericevic B, Conneely OM (2006). Overlapping and distinct expression of progesterone receptors A and B in mouse uterus and mammary gland during the estrous cycle.. Endocrinology.

[r19] Naciff JM, Overmann GJ, Torontali SM, Carr GJ, Khambatta ZS, Tiesman JP (2007). Uterine temporal response to acute exposure to 17α-ethinyl estradiol in the immature rat.. Toxicol Sci.

[r20] Nettles KW, Bruning JB, Gil G, O’Neill EE, Nowak J, Guo Y (2007). Structural plasticity in the oestrogen receptor ligand-binding domain.. EMBO Rep.

[r21] Nilsson S, Mäkelä S, Treuter E, Tujague M, Thomsen J, Andersson G (2001). Mechanisms of estrogen action.. Physiol Rev.

[r22] Piyachaturawat P, Ercharuporn S, Suksamrarn A. (1995). Uterotrophic effect of *Curcuma comosa* in rats.. Int J Pharmacogn.

[r23] Shifren JL, Schiff I (2010). Role of hormone therapy in the management of menopause.. Obstet Gynecol.

[r24] Su A, Sripanidkulchai K, Suksamrarn A, Hu Y, Piyachuturawat P, Sripanidkulchai B. (2012). Pharmacokinetics and organ distribution of diarylheptanoid phytoestrogens from *Curcuma comosa* in rats.. J Nat Med.

[r25] Suksamrarn A, Ponglikitmongkol M, Wongkrajang K, Chindaduang A, Kittidanairak S, Jankam A (2008). Diarylheptanoids, new phytoestrogens from the rhizomes of *Curcuma comosa*: Isolation, chemical modification and estrogenic activity evaluation.. Bioorg Med Chem.

[r26] Tibbetts TA, Mendoza-Meneses M, O’Malley BW, Conneely OM (1998). Mutual and intercompartmental regulation of estrogen receptor and progesterone receptor expression in the mouse uterus.. Biol Reprod.

[r27] Tora L, White J, Brou C, Tasset D, Webster N, Scheer E (1989). The human estrogen receptor has two independent nonacidic transcriptional activation functions.. Cell.

[r28] Wärnmark A, Treuter E, Gustafsson JA, Hubbard RE, Brzozowski AM, Pike AC (2002). Interaction of transcriptional intermediary factor 2 nuclear receptor box peptides with the coactivator binding site of estrogen receptor α.. J Biol Chem.

[r29] Winuthayanon W, Hewitt SC, Orvis GD, Behringer RR, Korach KS (2010). Uterine epithelial estrogen receptor α is dispensable for proliferation but essential for complete biological and biochemical responses.. Proc Natl Acad Sci USA.

[r30] Winuthayanon W, Piyachaturawat P, Suksamrarn A, Ponglikitmongkol M, Arao Y, Hewitt SC (2009a). Diarylheptanoid phytoestrogens isolated from the medicinal plant *Curcuma comosa:* Biological actions *in vitro* and *in vivo* indicate ER-dependent mechanisms.. Environ Health Perspect.

[r31] Winuthayanon W, Suksen K, Boonchird C, Chuncharunee A, Ponglikitmongkol M, Suksamrarn A (2009b). Estrogenic activity of diarylheptanoids from *Curcuma comosa* Roxb. requires metabolic activation.. J Agric Food Chem.

