# Supplementary material for: The Natural Estrogenic Compound Diarylheptanoid (D3): In Vitro Mechanisms of Action and in Vivo Uterine Responses via Estrogen Receptor α
Source: Environ Health Perspect. 2013 Jan 18;121(4):433–9. doi: 10.1289/ehp.1206122 (PMC3620745; doi:10.1289/ehp.1206122)
Supplement: (360 KB) PDF [file ehp.1206122.s001.pdf]

Supplemental Material

The Natural Estrogenic Compound Diarylheptanoid (D3): *In Vitro* Mechanisms of Action and *in Vivo* Uterine Responses via Estrogen Receptor α

Wipawee Winuthayanon, Pawinee Piyachaturawat, Apichart Suksamrarn, Katherine A. Burns, Yukitomo Arao, Sylvia C. Hewitt, Lars C. Pedersen, Kenneth S. Korach

Table of Contents

Figure S1 2

Supplemental Methods 3

References 5

Figure S1

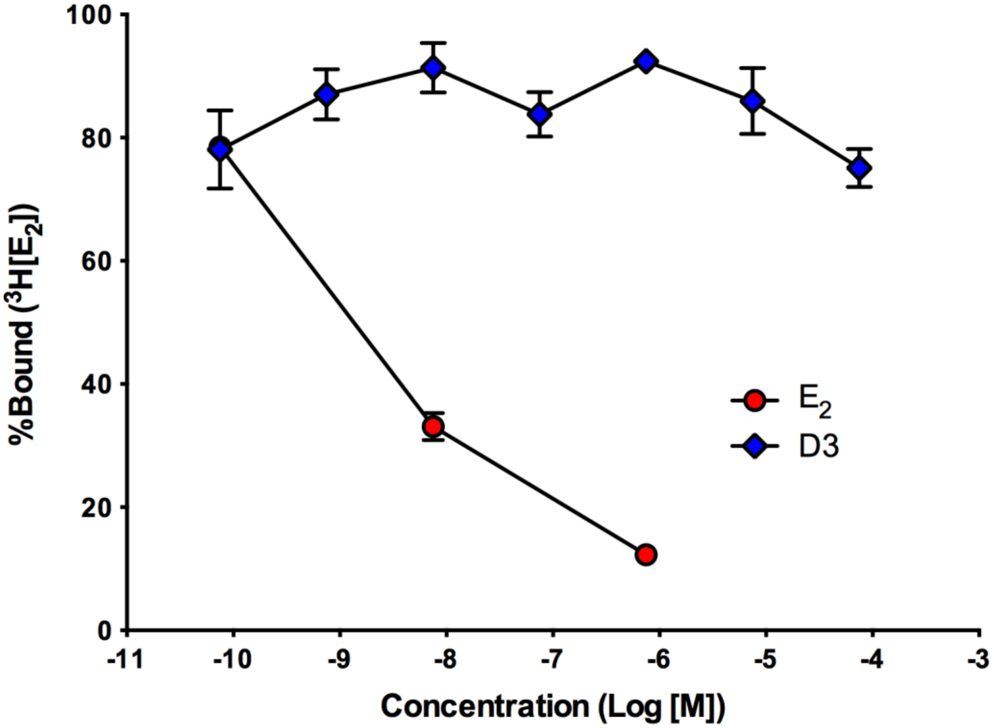

Figure S1: Competitive binding assay using unlabeled ligands, E_2 or D3, to displace $^3H[E_2]$ from estrogen receptor (ER) in uterine cytosol extract. Uterine cytosolic preparation was incubated with 7.5 nM $^3H[E_2]$ in the presence of indicated concentrations of E_2 (7.5×10^{-11} to 7.5×10^{-7} M) or D3 (7.5×10^{-11} to 7.5×10^{-5} M). Each value was obtained from three independent experiments performed in triplicate and presented as mean ± S.E.M.

Supplemental Methods

Cell transfection conditions. Ishikawa cells were plated in 24-well plates at a density of 8×10^5 cells/well in phenol red-free DMEM/F12 medium containing 10% dextran-coated charcoal treated fetal bovine serum or stripped FBS (SFBS; Hyclone, South Logan, UT, USA) and incubated an additional 32 h. Then, cells were transfected for 24 h with 0.1 μg of pcDNA3-containing designated ERα (or pcDNA3-empty vector as a control), 3×ERE-TATA-Luc, and pRL-TK by using Lipofectamine 2000 (Invitrogen). Cells were then treated with the test compounds for 24 h. The renilla and luciferase activities from the cell lysates were assayed using a Dual-Luciferase Reporter System (Promega) on LMAXII luminometer (Molecular Devices, Sunnyvale, CA, USA) according to the manufacturer's protocol.

Tissue collections. The uteri were collected, trimmed of fat, then blotted and weighed. A portion of each uterus was immediately frozen in liquid nitrogen for later RNA isolation with Trizol reagent (Invitrogen) according to the manufacturer's protocol. The remaining uterine samples were fixed in 10% formalin and embedded in paraffin and cross-sectioned. Immunohistochemistry of progesterone receptor (PR) and DNA incorporation of the EdU were analyzed as described previously (Winuthayanon et al. 2010).

Real-Time PCR. cDNA from mouse uteri was synthesized and cycle threshold (Ct) values were obtained by real-time PCR with SYBR Green I dye using the ABI PRISM 7900 Sequence Detection System and analysis software (Applied Biosystems, Foster City, CA, USA) as previously described (Hewitt et al. 2005). Applied Biosystems Primer Express Software version 2.0 was used for primer sequence generation. All primer sets were purchased from Sigma-Genosis (St. Louis, MO, USA). The primer sequences for *Aurkb* (aurora kinase B), *Ccnb2* (cyclin B2), *Fos* (FBJ osteosarcoma oncogene), *Inhbb* (inhibin beta-B), and *Rpl7* (ribosomal

protein L7) are as described (Hewitt and Korach 2011). The expression level was normalized to *Rpl7*, and an expression ratio was calculated relative to WT Veh control.

Competitive Binding Assay. Mice were obtained and handled as indicated in the Materials and Methods section. Uteri were removed from the adult ovariectomized C57BL6J mice (11-weeks-old),pooled and homogenized in ice-cold TEGM buffer (10 mM Tris-HCl, 1.5 mM EDTA, 10% Glycerol, 3.0 mM $MgCl_2$, pH 7.6) at 1 mL/50 mg tissue. The homogenates were then centrifuged for 50 min at 105,000 × *g*, and the supernatant, constituting the cytosol, was used for competitive binding assays. Cytosolic fractions were incubated with or without unlabeled ligands at designated concentrations for 1 h at 30ºC. Then, 7.5 nM of ^3H estradiol ([2,4,6,7-^3H(N)] estradiol ($^3H[E_2]$), PerkinElmer, Santa Clara, CA, USA.), was added and incubation was continued for an additional 30 min (Korach 1979). A slurry of 60% hydroxylapatite (Bio-Rad, Hercules, CA, USA) in TEGM buffer was added to adsorb the estrogen receptor (Erdos et al. 1970), and was washed twice with TEGM buffer to remove unbound ligand and then counted using Ecoscint A scintillation fluid (National Diagnostics, Atlanta, GA, USA) in a scintillation counter (Beckman Coulter, Fullerton, CA, USA). The percentage of bound $^3H[E_2]$ with or without the competing unlabeled ligands was determined by the following equation: % Bound $^3H[E_2]$ =100 × (counts per minute (CPM) $^3H[E_2]$ bound without the unlabeled ligand competition/ CPM $^3H[E_2]$ following the designated unlabeled ligand competition.

References

Erdos T, Best-Belpomme M, Bessada R. 1970. A rapid assay for binding estradiol to uterine receptor(s). Anal Biochem 37:244-252.

Hewitt SC, Collins J, Grissom S, Deroo B, Korach KS. 2005. Global uterine genomics in vivo: microarray evaluation of the estrogen receptor alpha-growth factor cross-talk mechanism. Mol Endocrinol 19:657-668.

Hewitt SC, Korach KS. 2011. Estrogenic activity of bisphenol A and 2,2-bis(p-hydroxyphenyl)-1,1,1-trichloroethane (HPTE) demonstrated in mouse uterine gene profiles. Environ Health Perspect 119:63-70.

Korach KS. 1979. Estrogen action in the mouse uterus: characterization of the cytosol and nuclear receptor systems. Endocrinology 104:1324-1332.

Winuthayanon W, Hewitt SC, Orvis GD, Behringer RR, Korach KS. 2010. Uterine epithelial estrogen receptor alpha is dispensable for proliferation but essential for complete biological and biochemical responses. Proc Natl Acad Sci U S A 107:19272-19277.
